# Unveiling urinary extracellular vesicle mRNA signature for early diagnosis and prognosis of bladder cancer

**DOI:** 10.7150/thno.107213

**Published:** 2025-01-01

**Authors:** Ning Sun, Zhaowei Zhang, Xiaoqing Yang, Jingqi Li, Qiang Li, Jingjing Kang, Yongchun Wei, Xiaoxuan Yu, Rui Du, Xiaoqin Hong, Guangming Liu, Hongmei Gao, Dingbin Liu

**Affiliations:** 1 State Key Laboratory of Medicinal Chemical Biology, Tianjin Key Laboratory of Molecular Recognition and Biosensing, Frontiers Science Center for New Organic Matter, College of Chemistry, Nankai University, Tianjin 300071, China.; 2 Tianjin Institute of Urology, the Second Hospital of Tianjin Medical University, Tianjin 300211, China.; 3 Department of Urology, Tianjin First Center Hospital, Nankai University, Tianjin 300071, China.; 4 Department of Intensive Care Unit, Key Laboratory for Critical Care Medicine of the Ministry of Health, Emergency Medicine Research Institute, Tianjin First Center Hospital, Nankai University, Tianjin 300071, China.

**Keywords:** bladder cancer, extracellular vesicle, urine, mRNA, biomarker

## Abstract

**Background:** Bladder cancer (BC) ranks as one of the most prevalent cancers. Its early diagnosis is clinically essential but remains challenging due to the lack of reliable biomarkers. Extracellular vesicles (EVs) carry abundant biological cargoes from parental cells, rendering them as promising cancer biomarkers. Herein, we revealed that urinary EVs (uEVs) mRNA signature could serve as non-invasive biomarker for the early diagnosis and prognostic assessment for BC.

**Methods:** Transcriptomic sequencing was conducted to reveal the mRNA signature of EVs collected from normal cell line and different grades of BC cell lines. Candidate EV-mRNA biomarkers were further profiled using clinical urine samples (*n* = 97, including healthy controls, BC patients and post-surgery BC patients) by RT-qPCR.

**Results:** Three mRNAs (SRGN, FLI1, and MACROH2A2) within uEV were identified as potential biomarkers for BC, providing an area under the receiver operating characteristic curve (AUC) of 0.973 for BC diagnosis. Moreover, the uEV-mRNA panel could discriminate early-stage BC patients from healthy controls with an AUC of 0.969. Finally, we found that the uEV-mRNAs were significantly down-regulated in the post-surgery urine samples of BC patients.

**Conclusions:** Given the facile and non-intrusive nature of urine collection, the identified uEV-mRNAs could serve as potential liquid-biopsy biomarkers for the early diagnosis and prognosis of BC.

## Introduction

Bladder cancer (BC) represents one of the most common malignancies, with approximately 614,000 incidences and 220,000 fatalities occurring in 2022 1,2. BC is generally stratified into two primary forms: muscle-invasive bladder cancer (MIBC) and non-muscle-invasive bladder cancer (NMIBC), as delineated by different clinical manifestations 3. In the clinic, 75% of diagnosed individuals exhibit as NMIBCs, with a typical five-year survival rate of 90% 4. In contrast, the remaining 25% cases manifest as MIBCs and have a poorer prognosis, showing a five-year survival rate between 20% and 40%. The five-year survival rate for BC has rarely been improved since 1990s 5. More dishearteningly, nearly half of MIBC cases are prone to metastasize within three years after diagnosis 6. These facts underscore the urgent need for a discerning diagnostic modality for early diagnosis and real-time monitoring of BC progression.

Hematuria stands prominently as a typical symptom closely linked to BC, making it an indicator for BC assessment in a majority of patients 7. The absence of hematuria in patients corresponds to an extended interval between manifestation of symptoms and conclusive diagnosis. Furthermore, the intermittent nature of hematuria significantly diminishes the informative value of test strip screening, even within cohorts at heightened risk, such as heavy smokers and those enduring prolonged exposure to carcinogens 8. Presently, cystoscopy assumes a pivotal role as the primary tool for BC screening and remains the gold standard for diagnosing BC. However, cystoscopy could involve the risk of infection due to its invasiveness and may provide controversial results particularly when diagnosing the patients devoid of hematuria 8,9. Although urine cytology is commonly used for BC diagnosis, its utility remains limited because of its low sensitivity and reproducibility 10,11. While molecular imaging techniques offer substantial support in identifying malignant BC lesions and staging, their ionizing radiation property as well as high expense have hampered their capacity to effectively monitor tumor recurrence and metastasis 12. Consequently, an imperative emerges for discovering biomarkers with high clinical specificity and sensitivity adaptable to the diagnosis of BC.

Extracellular vesicles (EVs) are cell-derived nano-sized phospholipid membrane-enclosed structures 13 that could be secreted into diverse body fluids—urine 14,15, blood 16,17, tear 18,19, and saliva 20,21, etc. EVs have attracted widespread attention in biomedical field because they inherit specific molecular information from their parental cells 22-25. In particular, urine-derived EVs (namely uEVs) have emerged as promising biomarkers for urinary tract diseases owing to their close relationship and the non-invasive way to obtain urine samples 15,26. It is noteworthy that free mRNAs in urine tend to be degraded rapidly by ribonuclease (RNase). EV-encapsulated mRNAs, on the contrary, could maintain their structural integrity owing to the protection afforded by the bilayer phospholipid structure of EVs 27,28. Hence, analysis of uEV-mRNA expression profiles may provide new opportunities for the identification of potential biomarkers for BC diagnosis.

In this study, we conducted a comprehensive analysis of RNA expression profiles in both BC and normal cells using transcriptomic sequencing. Through this analysis, we identified a panel of EV-wrapped diagnostic mRNA biomarkers including SRGN, FLI1, and MACROH2A2 that are specific to BC, subsequently confirming their validity through experimentation with human urine samples (*n* = 97). Furthermore, we investigated the potential of these biomarkers for the early detection and prognostic assessment of BC, where they showed high clinical sensitivity and specificity.

## Methods

### Cell Culture

Six cell lines were purchased from the National Collection of Authenticated Cell Cultures (Shanghai, China). Normal human bladder urothelial cells (SVHUC1) were cultured in Ham's F-12K medium supplemented with 10% (v/v) fetal bovine serum (FBS, Gibco) and 1% (v/v) penicillin-streptomycin (PS, Gibco) in an incubator under 5% CO_2_ at 37 °C. RT4 was cultured in McCoy's 5A medium (Gibco) supplemented with 10% (v/v) FBS (Gibco) and 1% (v/v) PS (Gibco) in an incubator under 5% CO_2_ at 37 °C. The HK-2, 5637, T24, and EJ cells were cultured in RPMI-1640 medium (Gibco) supplemented with 10% (v/v) FBS (Gibco) and 1% PS (v/v) (Gibco) in an incubator under 5% CO_2_ at 37 °C. An appropriate volume of serum-free medium was added until the cells grew to 70-80% density. After 48 hours, the supernatant was collected for EVs isolation.

### Isolation of the EVs from culture medium

EVs were isolated from the collected culture medium by centrifugation and ultracentrifugation. The collected cell culture medium was centrifuged at 300 g for 10 min and 2000 g for 30 min, filtered through a 0.22 µm filter membrane, and continuously centrifuged at 10,000 g for 30 min to remove dead cells, cell debris, and large contaminating vesicles. Next, 420 mL of the medium was ultracentrifuged at 110,000 g for 70 min. The pellet was resuspended in PBS (pH 7.2) and ultracentrifuged at 110,000 g for 70 min again (Type 45 Ti rotor, Beckman). The final EV pellet was resuspended in 100 µl fresh prechilled PBS (pH 7.2) and stored in a -80 °C freezer for future use. All centrifugations were carried out at 4 °C.

### Collection of clinical urine samples

Midstream urine samples from 69 BC patients and 28 healthy volunteers were obtained in the morning and kept at 4 °C. HC were collected from healthy donors who went through a medical check-up. Urine was collected after signing the informed consent, and the study was approved by the Science and Technology Ethics Committee of Tianjin First Central Hospital (No. 2023DZX12). In all cases, the urine samples were centrifuged in the sterile tubes within 8 h of collection at 2000 g for 30 min at 4 °C to remove cells and large cell debris and then frozen at -80 °C in time.

### Isolation of uEVs

A volume of 30-50 mL of urine samples from donors was thawed at 4 °C and vortexed for 60 s. Subsequently, the urine was centrifuged at 8000 g for 30 min and filtered through a 0.22 µm filter membrane. Next, the sample was ultracentrifuged at 100,000 g for 90 min (Type 45 Ti rotor, Beckman). Supernatants were removed, and the pellet was washed in 70 ml of PBS (pH 7.2), repeating the same ultracentrifugation. Finally, uEVs were resuspended in 50 µl fresh prechilled PBS (pH 7.2) and stored in a -80 °C freezer for future use. All centrifugations were carried out at 4 °C.

## Characterization of EVs

### Morphology characterization

The micromorphology of EVs was observed by a 200 kV cryo-transmission electron microscope (cryo-TEM) (Talos F200C, ThermoFisher Scientific, USA). Each EV sample was stained with 1% uranium dioxide acetate solution and dropped onto a copper grid.

### Nanoparticle tracking analysis (NTA)

The size of EVs was measured by NTA using a Malvern NanoSight NS300 instrument. The EVs were diluted in 1 mL PBS (pH 7.2) and mixed well, and then the EV particle sizes were recorded and analyzed by NTA software (version 3.4, NanoSight).

### Western blot (WB) analysis

EVs were lysed with RIPA buffer containing 1 mM PMSF on ice for 30 min and continued by centrifugation at 12,000 g at 4 °C for 10 min. The protein amount was quantified using a BCA kit. Subsequently, the lysates were mixed with 1×loading buffer at a final concentration and heated at 95 °C for 10 min. After leaving at room temperature for 5 min, the samples were separated by SDS-PAGE and transferred onto a 0.45 µm nitrocellulose membrane at 4 °C. Then the membranes were blocked with 5% (w/v) skim milk powder (dissolved in TBST buffer: 20 mM Tris-HCl, 150 mM NaCl, and 1% Tween-20) for 35 min at room temperature and incubated with primary antibodies (1:1000): Anti-CD9, Anti-CD63, Anti-TSG101 at 4°C for 12 h, followed by washing three times with TBST buffer. The membranes were incubated with secondary antibodies (HRP-Goat anti-rabbit IgG, 1:1000) for 1.5 h at room temperature, followed by washing three times with TBST buffer. Finally, the membranes were displayed by using chemiluminescent reagent (BeyoECL Plus) for 1 min and exposed by a gel image instrument (Azure c600, Azure Biosystems, USA). All the antibodies were obtained from Beyotime Biotechnology (Shanghai, China).

### Transcriptome sequencing and bioinformatics analysis

A total of 1 mg RNAs were extracted from EVs isolated from six cell lines using TRIzol reagents (Invitrogen, Carlsbad, CA, USA). RNA-seq and data analysis were performed by LC-Bio Technology CO. (Zhejiang, China). The genes with fold change (FC) > 1.5 and p-value < 0.05 were used to define DEGs, followed by GO and KEGG enrichment analysis. Protein-protein interaction (PPI) network of DEGs was analyzed by STRING (https://cn.string-db.org/), and the top ten hub genes from PPI were performed using Cytoscape software.

### RNA extraction

Total RNA was extracted from EVs derived from cells and human urine using the UNIQ-10 Column TRIzol Total RNA Extraction Kit (Sangon Biotech, China). Finally, the extracted RNA was dispersed in 35 µl of DEPC-treated ddH_2_O. The yield and quality of RNAs were analyzed by the microplate reader (Bio Tek).

### RT-qPCR analysis

Total RNA was used to synthesize the first strand cDNA using the MightyScript first chain cDNA synthesis Master Mix (**Table S5**). 2× II M-MLV RT Mix was added to the appropriate concentration of RNA solution in ice to obtain a mixture. Subsequently, the mixture was incubated at room temperature for 5 min and reverse-transcribed at 42 °C for 30 min and at 85 °C for 5 min to inactivate the reverse transcriptase. The amplification reactions and detection were performed using a LightCycler 96 instrument (Roche, Switzerland) and carried out with SGExcel FastSYBR qPCR Mixture according to the manufacturer's protocol (Sangon Biotech, China). The standard temperature profile was shown in **Table S6**. The GAPDH was selected as the internal reference to analyze the relative expression of target genes. The gene was considered unexpressed when the Cq value was greater than 38.

### Statistical analysis

The comparisons between different groups of EVs isolated from cell lines and urine samples were assessed using an unpaired *t*-test and Mann-Whitney test, respectively. Pre- and post-surgery sample test results were assessed using paired *t*-test. Statistical analyses were performed using GraphPad Prism (version 9.3.1). Data from the experiments were expressed as mean ± SD based on three independent experiments. A p-value < 0.05 was considered statistically significant. The logistic regression analysis was used to combine mRNAs into one marker by IBM SPSS Statistics (version 26.0). The confusion matrix was performed using MATLAB software (MATLAB, R2021b). ROC curves, AUC, sensitivity, specificity, and 95% CIs calculation were also performed by GraphPad Prism (version 9.3.1).

## Results

### Study design

**Figure 1:**
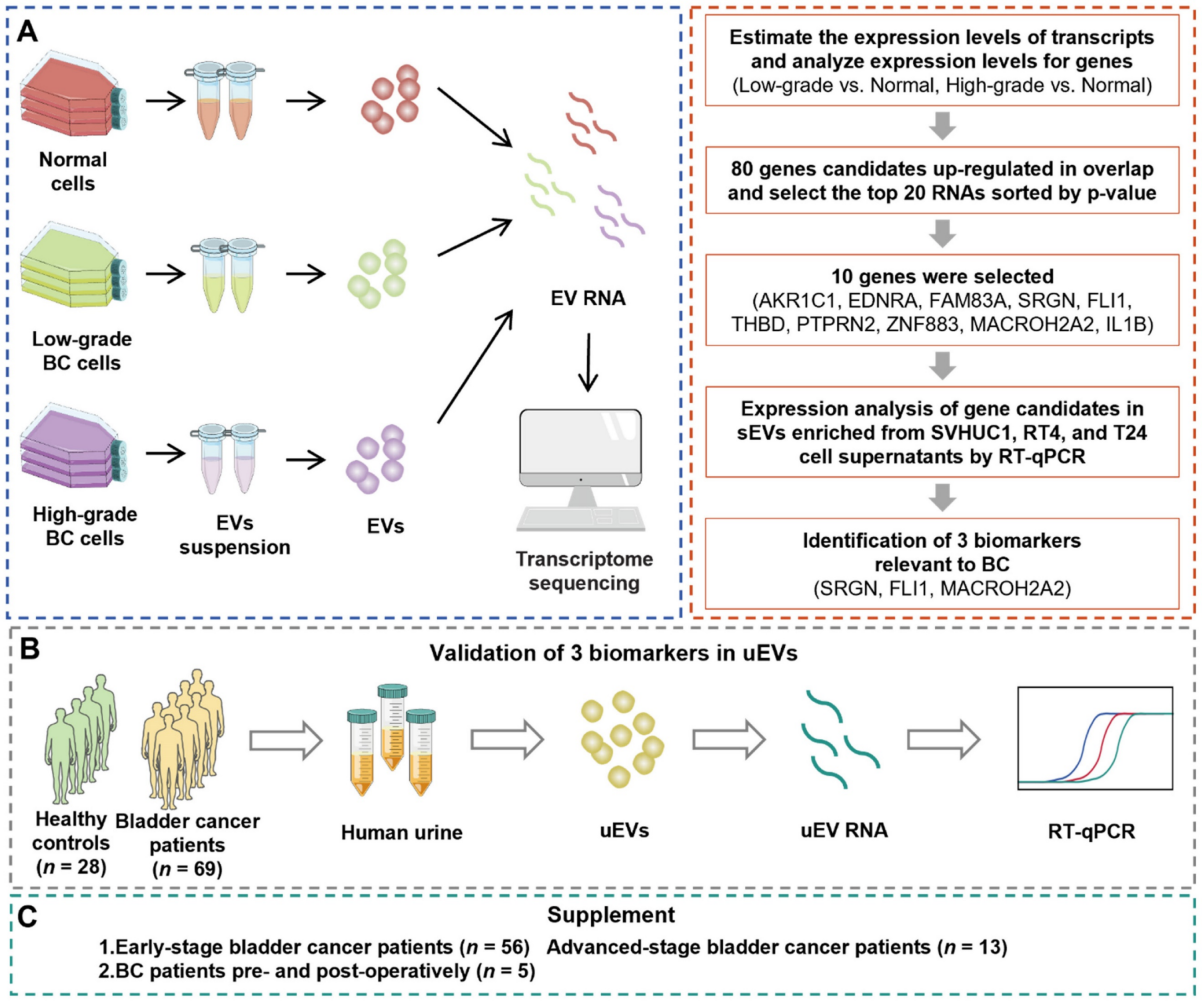
** The overall workflow of the study.** (**A**) The discovery phase: systematic analysis involved transcriptomic profiling of EVs obtained from the cell lines: normal cells (SVHUC1, HK-2), low-grade BC cells (5637, RT4), and high-grade BC cells (T24, EJ). Then, candidate genes were screened by RT-qPCR, from which SRGN, FLI1, and MACROH2A2 in EVs were identified for the subsequent investigations. (**B**) The validation phase: uEVs isolated from both healthy (*n* = 28) and BC (*n* = 69) urine samples to affirm the diagnostic efficacy of SRGN, FLI1, and MACROH2A2. (**C**) The supplementary phase: uEVs obtained from healthy cohort and BC patients with different stages, as well as those from pre- and post-surgery BC patients, were employed to assess the ability of SRGN, FLI1, and MACROH2A2 for early diagnosis and prognosis of BC.

We performed this study with a 3-phase strategy to uncover mRNA biomarkers that are capable of accurately distinguishing BC patients from healthy control (HC) cohort (**Figure 1**). During the primary discovery phase, a systematic analysis involved transcriptomic profiling of EVs originated from various cell lines: normal cells (SVHUC1, HK-2), low-grade BC cells (5637, RT4), and high-grade BC cells (T24, EJ). RNA profiles were utilized to conduct a differential expression analysis of EVs derived from low-grade BC cells and normal cells, as well as that between high-grade BC cells and normal cells. Subsequently, mutual differentially expressed genes (DEGs) from both sets were identified for further experiments. RT-qPCR was conducted to evaluate the candidate gene expression levels within the cellular EVs. Ultimately, three discerning biomarkers were identified for the subsequent studies. The following validation phase involved utilization of uEVs isolated from both BC and healthy urine samples to affirm the diagnostic efficacy of the identified biomarkers from the discovery phase. RT-qPCR assays were employed to assess the candidate gene expression levels within uEVs. During the final supplementary phase, uEVs obtained from individuals representing the HC cohort, early-stage and advanced-stage BC patients, as well as those from pre- and post-surgery BC patients, were employed to assess the biomarkers' ability for early-stage BC detection and prognostic potential for BC.

### Isolation and characterization of cell-derived EVs

**Figure 2:**
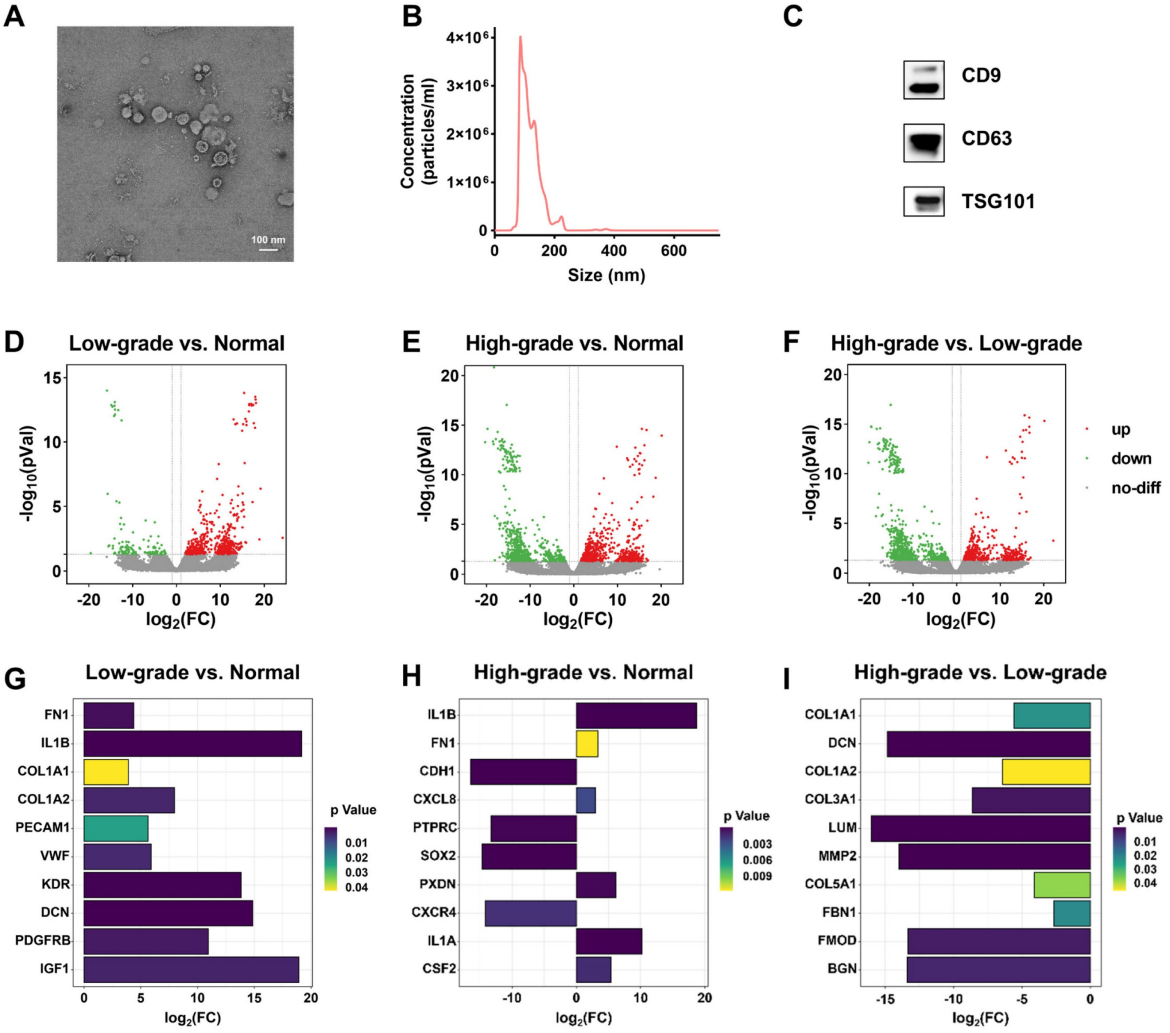
** Characterization and transcriptomic analysis of BC-related EVs.** Characterization of EVs derived from T24 cells by (**A**) cryo-TEM, (**B**) NTA, and (**C**) WB. Transcriptomic profiling of EVs derived from normal cells (SVHUC1, HK-2), low-grade BC cells (5637, RT4), and high-grade BC cells (T24, EJ). Volcano plot of differentially expressed genes (DEGs) in EVs derived from (**D**) low-grade BC cells relative to normal cells, (**E**) high-grade BC cells relative to normal cells, and (**F**) high-grade BC cells relative to low-grade cells. The differential expression degrees of hub genes in the EVs derived from (**G**) low-grade BC cells relative to normal cells, (**H**) high-grade BC cells relative to normal cells, and (**I**) high-grade BC cells relative to low-grade cells.

To obtain BC-associated EVs, we used ultracentrifugation to enrich EVs from six types of cells, including high-grade BC cells (T24 and EJ), low-grade BC cells (RT4 and 5637), and normal cells (SVHUCI and HK-2). T24 cell-derived EVs were characterized as an example by cryo- transmission electron microscope (cryo-TEM), nanoparticle tracking analysis (NTA), and western blot (WB) analysis. Cryo-TEM showed the typical saucer-shaped structure of EVs (**Figure 2A**). NTA was used to characterize the particle size of EVs as 125.2 ± 1.7 nm (**Figure 2B**). Finally, WB analysis validated the presence of general markers (CD9, CD63, and TSG101) of EVs. (**Figure 2C**).

### Transcriptomic profiling

To screen EV biomarkers for BC, three groups of EVs isolated from the supernatants of normal, low-grade BC, and high-grade BC cell lines were subjected to transcriptomic analysis. The results show that, relative to normal cell-derived EVs, 494 DEGs were significantly up-regulated and 120 DEGs were significantly down-regulated in high-grade BC cell-derived EVs; 657 DEGs were significantly up-regulated and 754 were significantly down-regulated in low-grade BC cell-derived EVs. Simultaneously, 527 significantly up-regulated DEGs and 938 significantly down-regulated DEGs were found in high-grade BC cell-derived EVs relative to low-grade BC cell-derived EVs (**Figure S1**). **Figure S2** summarizes the gene expression levels of six EV samples. We next performed cluster analysis among the three groups of EV samples and found that each group had a different spectrum (**Figure S3**). However, there are similar profiles between SVHUC1 and HK-2, 5637 and RT4, and T24 and EJ at the same BC grade as the cell lines, indirectly indicating the reliability of the sequencing results. The volcano plots (**Figure 2D-F**) for the three groups of EV samples were also significantly different. Compared with low-grade BC cell-derived EVs, EVs derived from high-grade BC cell exhibited more significant differences in genetic expression relative to normal cell-derived ones.

Gene ontology (GO) analysis in **Figure S4** and **Figure S5** shows that the DEGs obtained from the BC cell-derived EVs versus normal cell-derived EVs are mainly involved in the biological processes of signal transduction, cell adhesion and multicellular organism development that controls the molecular functions of protein binding and metal ion binding. Signal transduction is associated with many diseases, including cancer, while cell adhesion is closely related to tumor invasion and metastasis. Therefore, potential biomarkers or relevant therapeutic targets can be identified from the genes categorized into this function, such as plexin family genes 29,30 and collagen family genes 31-33. Furthermore, Kyoto Encyclopedia of Genes and Genomes (KEGG) analysis in **Figure S6** and **Figure S7** shows that the pathway in cancer had the largest number of DEGs in all BC cell-derived EVs, implying that these DEGs could be served as candidate BC biomarkers and relevant therapeutic targets.

In addition, we analyzed the protein-protein interaction (PPI) network of DEGs between the three groups (**Figure S8** and **Figure S9**), from which we identified the top ten hub genes of each group (**Figure S10**). The hub genes in the group of low-grade BC cell-derived EVs versus normal cell-derived EVs include fibronectin 1 (FN1) 34, interleukin 1 beta (IL1B) 35, collagen type I alpha 1 chain (COL1A1) 36, collagen type I alpha 2 chain (COL1A2) 37, platelet and endothelial cell adhesion molecule 1 (PECAM1) 38, von Willebrand factor (VWF) 39, kinase insert domain receptor (KDR) 40, decorin (DCN) 41, platelet-derived growth factor receptor beta (PDGFRB) 42, and insulin like growth factor 1 (IGF1) 43,44, all of which were significantly upregulated (**Figure 2G**). The significantly upregulated hub genes in the group of high-level BC cell-derived EVs versus normal cell-derived EVs include IL1B, FN1, C-X-C motif chemokine ligand 8 (CXCL8) 45, peroxidasin (PXDN) 46, interleukin 1 alpha (IL1A) 47, and colony stimulating factor 2 (CSF2) 48 (**Figure 2H**). We noted that the hub genes were all downregulated in the group of high-grade BC cell-derived EVs versus low-grade BC cell-derived EVs (**Figure 2I**). It has been shown that these upregulated hub genes are associated with many cancer risks and may serve as potential biomarkers or therapeutic targets for BC, especially FNI and IL1B, which were upregulated in the expression of both low-grade and high-grade BC cell-derived EVs.

### Potential biomarker screening from the sequencing results and validation with RT-qPCR

**Figure 3:**
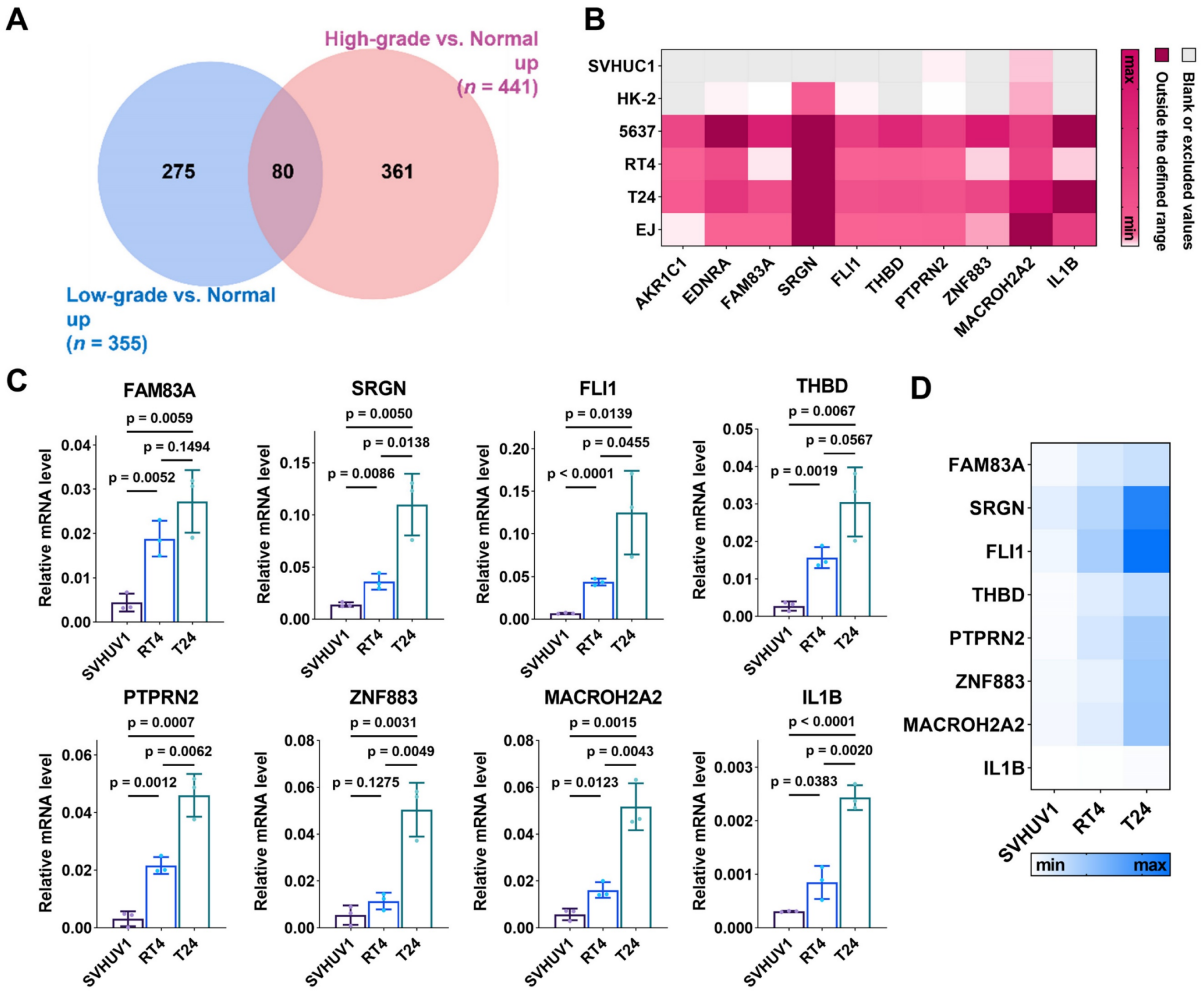
** Discovery of candidate BC biomarkers.** (**A**) Venn diagram of significantly upregulated genes in EVs derived from low-grade BC cells and high-grade BC cells relative to those derived from normal cells. (**B**) Expression heatmap of the 10 gene panel with EVs derived from SVHUC1, HK-2, 5637, RT4, T24, and EJ cells from sequencing results. Scattering plots (**C**) and heatmap (**D**) of the relative expression levels of EV-RNAs derived from SVHUC1, RT4 and T24 cells. Relative mRNA level was analyzed by RT-qPCR.

To obtain potential biomarkers for BC, we focused on the DEGs with expression > 0 and upregulated in both high-grade and low-grade BC cell-derived EVs relative to normal cell-derived EVs. In the sequencing results, 441 and 355 genes were respectively up-regulated in high-grade and low-grade BC cell-derived EVs relative to normal cell-derived EVs, with 80 overlapping genes (**Figure 3A**). Among these 80 genes, we selected top ten genes with the highest degree of up-regulation as candidate biomarkers, including aldo-keto reductase family 1 member C1 (AKR1C1), endothelin receptor type A (EDNRA), family with sequence similarity 83 member A (FAM83A), serglycin (SRGN), friend leukemia integration 1 (FLI1), thrombomodulin (THBD), protein tyrosine sequence phosphatase receptor type N2 (PTPRN2), zinc finger protein 883 (ZNF883), macroH2A.2 histone (MACROH2A2), and IL1B. These genes in the free form have been studied as potential cancer biomarkers or relevant therapeutic targets, whereas those in EVs have rarely been explored. AKR1C1 encodes a member of the aldehyde/ketone reductase superfamily, which is involved in cellular biosynthesis and metabolism. It induces tumor progression and metastasis 49. EDNRA encodes an endothelin-1 receptor that plays a role in effective and long-lasting vasoconstriction. It participates in the proliferation of cell populations, which is highly relevant to tumor progression 50. FAM83A is involved in cell proliferation and epidermal growth factor receptor signaling pathway. FAM83A is overexpressed in many human cancers 51. SRGN encodes a hematopoietic granule proteoglycan protein associated with apoptotic processes in the development of cellular tissues and is up-regulated in various cancers 52. FLI1 encodes a transcription factor that contains the DNA-binding domain of the E26 transforming-specific (ETS) family involved in cellular growth and angiogenesis, which induces tumor progression and metastasis 53. THBD encodes an endothelium-specific type I membrane receptor. PTPRN2 belongs to the protein tyrosine phosphatase family and is involved in cell migration and cancer metastasis 54. ZNF883 encodes a zinc finger protein that may be involved in transcriptional regulation. MACROH2A2 encodes a replication-independent histone that represses transcription and restricts cellular plasticity in differentiated and cancer cells 55. IL1B encodes a protein that is a member of the interleukin 1 cytokine family, which is involved in cell proliferation, cell apoptosis, and metabolism 56. In addition, IL1B involves in the development of various cancers 57.

Expression of the ten mRNAs selected above is depicted in **Figure 3B**, showing differentiated levels in EVs derived from six different cell lines. To validate these sequencing results, we extracted total RNA of EVs isolated from cell supernatants (SVHUC1, RT4 and T24 cells) and performed RT-qPCR. The primer sequences corresponding to the target genes are shown in **Table S1**. The formula 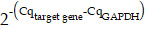 was used to calculate the relative expression of each target mRNA (**Figure 3C** and** 3D**). For all selected mRNAs, the expression levels were up-regulated with the increase of cellular BC grade, suggesting that these ten mRNAs are eminently potential to serve as BC diagnostic biomarkers. Nevertheless, the expression of several mRNAs (FAM83A, THBD, PTPRN2, ZNF883, and IL1B) in uEVs was too low to be detected in the subsequent validation cohort. To ensure the effective detection in human urine samples, we eventually chose SRGN, FLI1, and MACROH2A2 for the following study.

### BC diagnosis using SRGN, FLI1, and MACROH2A2 in uEVs

**Figure 4:**
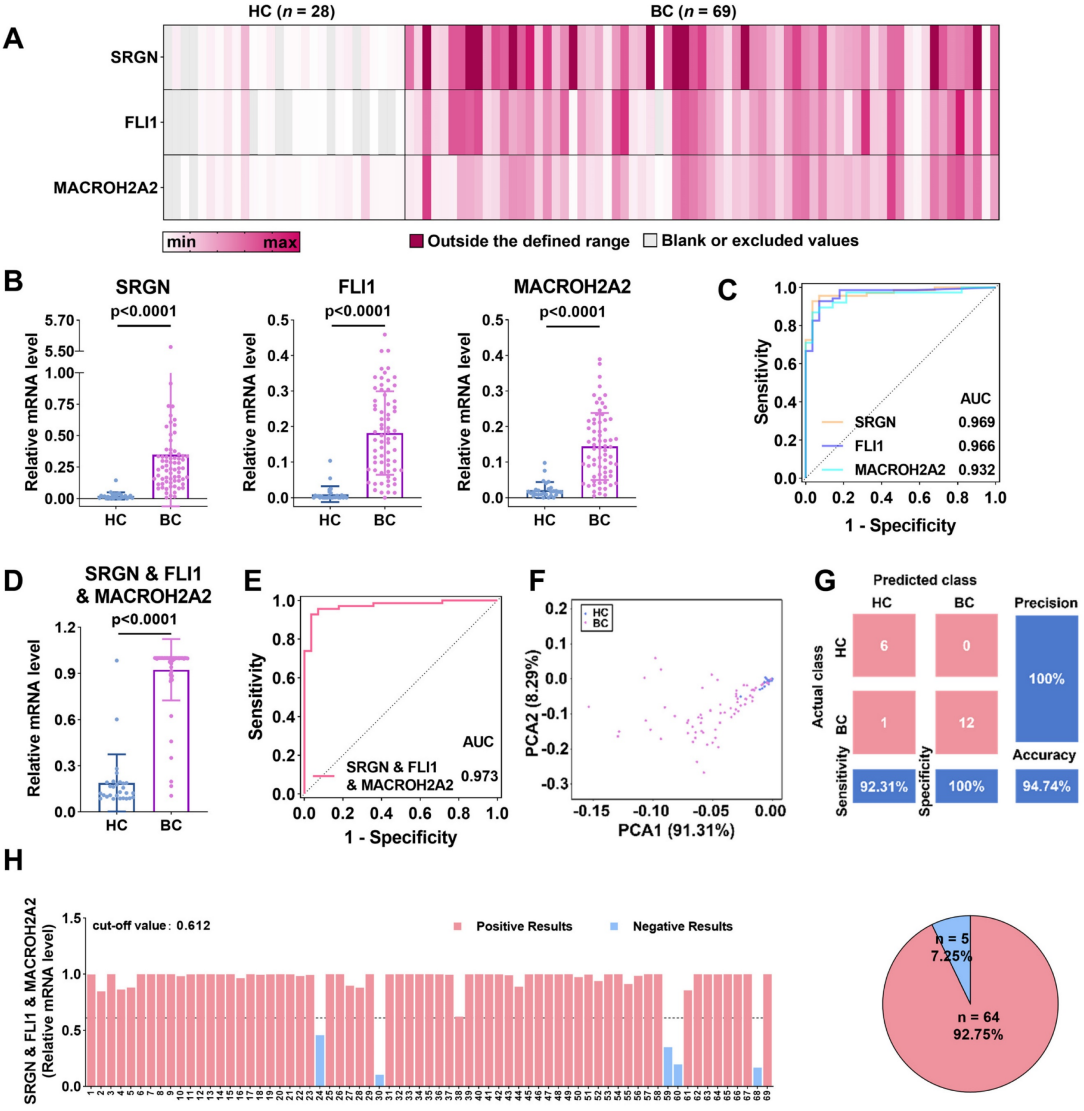
** BC diagnosis by SRGN, FLI1 and MACROH2A2 in uEVs.** (**A**) Heatmap of the three mRNAs in uEVs collected from HC (*n* = 28) and patients with BC (*n* = 69). (**B**) Scattering plots of the mRNA expression levels of SRGN, FLI1 and MACROH2A2 in uEVs collected from HC and patients with BC. (**C**) ROC curves of the individual markers for BC diagnosis. (**D**) Scattering plots of the mRNA expression levels of the weighted sum of the three markers in uEVs collected from HC and patients with BC. (**E**) ROC curves of the weighted sum calculated by logistic regression algorithm of the three markers for BC diagnosis. (**F**) PCA plot demonstrates the obvious separation in mRNA expression between HC and BC. (**G**) Confusion matrix of the weighted sum of three biomarkers. (**H**) BC subjects were divided into two subgroups of high and low mRNA expression levels by the weighted sum of the three markers. A cut-off value of 0.612 was determined by Youden index (YI). All statistical analyses were performed at 95% CIs.

Considering the potential differences between *in-vivo* and *in-vitro* conditions, we further evaluated the clinical performance of these three mRNAs in BC diagnosis. We collected urine samples from healthy controls (HCs, *n* = 28) and BC patients (*n* = 69), whose clinical information was provided in **Table S2**. The uEVs were subsequently isolated and characterized by cryo-TEM, NTA, and WB. These results are consistent with that of the EVs extracted from cell lines (**Figure S11**). As shown in the heatmap (**Figure 4A**), the average expression of SRGN, FLI1, and MACROH2A2 in uEVs derived from BC patients is evidently higher than those in the HC group. Scatter plots (**Figure 4B**) demonstrated that SRGN (p < 0.0001), FLI1 (p < 0.0001), and MACROH2A2 (p < 0.0001) were statistically significant as individual biomarkers in distinguishing BC patients from HC donors.

Accumulating evidence shows that the combination of multiple EV biomarkers may improve the diagnostic performance of diseases 58,59. Therefore, we combined these three biomarkers to further evaluate the diagnostic sensitivity and specificity for BC (**Figure 4D** and **Figure S12A**). Receiver operating characteristic (ROC) analysis was conducted to assess the performance of the three biomarkers and their combinations for BC diagnosis. The results indicated that the areas under the ROC curves (AUCs) of each combination were larger than those of the individual biomarkers (**Figure 4C, 4E** and **Figure S12B**). The optimal diagnostic performance was obtained by combining the three biomarkers into one marker with an AUC of 0.973 (95% CI: 0.944-1.000, sensitivity = 92.8%, specificity = 96.4%) (**Table S3**).

After combining the three biomarkers into one marker, we performed principal component analysis (PCA) to investigate the discrimination between the HC and BC patient groups (**Figure 4F**). The three-biomarker-combination was subjected to linear discriminant analysis (LDA) to differentiate the two groups, where 20% of the samples were randomly selected for validation. The results were presented as a confusion matrix (**Figure 4G**), with 92.31% sensitivity, 100% specificity, 94.74% accuracy, and 100% precision.

Finally, Youden index (YI) was utilized to establish the diagnostic cut-off values, which maximized the sum of sensitivity and specificity in ROC curves, and was subsequently applied to the independent diagnosis of BC 60. In details (**Figure 4H** and **Figure S13**), the cut-off values for SRGN, FLI1, and MACROH2A2 were set as 0.063, 0.029, and 0.030, respectively. Their diagnostic accuracy, correspondingly, was estimated to be 92.75%, 92.75%, and 91.30%. The cut-off values for the combinations of two biomarkers, *i.e.*, SRGN and FLI1, SRGN and MACROH2A2, and FLI1 and MACROH2A2, were set as 0.616, 0.596 and 0.514, respectively, and all of them had an accuracy of 92.75%. The cut-off value for the three-biomarker-combination was determined to be 0.612, slightly lower than that of the combination of SRGN and MACROH2A2, while the accuracy remained 92.75%. These results confirm that SRGN, FLI1, and MACROH2A2 could be served as potential biomarkers for BC diagnosis with high clinical performance.

### Early-stage BC diagnosis using SRGN, FLI1, and MACROH2A2 in uEVs

**Figure 5:**
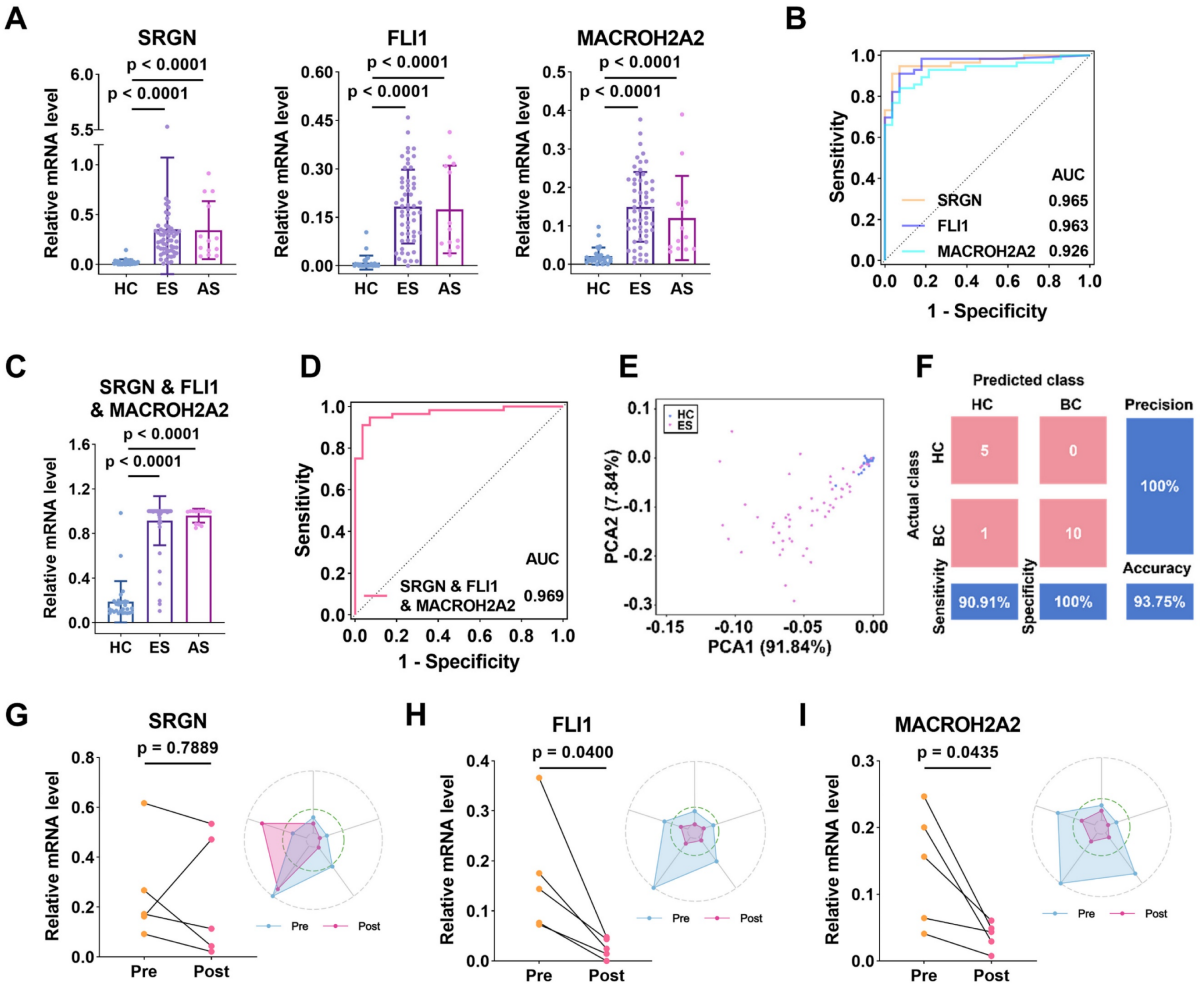
** Early diagnosis and prognosis of BC by SRGN, FLI1 and MACROH2A2.** (**A**) Scattering plots of the mRNA expression levels of SRGN, FLI1 and MACROH2A2 in uEVs collected from HC (*n* = 28), early-stage (ES) BC patients (*n* = 56), and advanced-stage (AS) BC patients (*n* = 13). (**B**) ROC curves of the individual markers for early-stage BC diagnosis. (**C**) Scattering plots of the mRNA expression levels of the weighted sum of the three markers in uEVs collected from HC, early-stage BC patients, and advanced-stage BC patients. (**D**) ROC curves of the weighted sum calculated by logistic regression algorithm of the three markers for early-stage BC diagnosis. (**E**) PCA plot demonstrates the obvious separation in mRNA expression between HC and early-stage BC. Sample 20 was not shown due to high expression level of SRGN. (**F**) Confusion matrix of the weighted sum of three biomarkers. (**G-I**) Longitudinal urine sample analysis of 5 patients undergoing surgery. All statistical analyses were performed at 95% CIs.

To assess the ability of the three uEV-mRNA biomarkers in early BC diagnosis, we used RT-qPCR to exclusively and independently analyze the expression levels of the three biomarkers in the uEVs collected from patients with early-stage BC. As shown in **Figure 5A**, the relative expression of SRGN (p < 0.0001), FLI1 (p < 0.0001), and MACROH2A2 (p < 0.0001) in uEVs from patients with early-stage BC (*n* = 56) was significantly higher than those in uEVs from HC (*n* = 28). Similarly, we also combined the three biomarkers as one single marker to evaluate the diagnostic performance. The three-biomarker-combination in uEVs of BC group at early stages also displayed significant elevation over HC group (p < 0.0001) (**Figure 5C**). The remaining combinations are shown in **Figure S14A**.

The capacity of SRGN, FLI1, and MACROH2A2 in distinguishing early-stage BC patients was reported by ROC curves (**Figure 5B**). The results showed that they also presented high clinical performance, with AUCs of 0.965, 0.963 and 0.926, respectively. The AUC of the three-biomarker-combination was further enhanced to 0.969 (95% CI: 0.9358-1.000, sensitivity = 94.6%, specificity = 92.9%) (**Figure 5D**), and the results of the remaining combination cases are displayed in **Figure S14B** and **Table S4**. The PCA plot of the three-mRNA-combination also showed a clear distinction between the HC group and the group of early-stage BC patients (**Figure 5E**). The categorization results between the two groups were also subjected to LDA, and 20% of the samples were randomly selected for validation. The confusion matrix showed a sensitivity of 90.91%, a specificity of 100%, an accuracy of 93.75%, and a precision of 100% (**Figure 5F**). The results above indicate that expressions of SRGN, FLI1, and MACROH2A2 in uEVs were significantly upregulated in the early-stage BC compared with HC. The combination of these three biomarkers demonstrated a high diagnostic efficiency for early-stage BC, thereby indicating a significant potential for clinical application.

### Prognostic capacity of SRGN, FLI1, and MACROH2A2 in BC patients

We also assessed the prognostic ability of these uEV-mRNA biomarkers by comparing the expressions of SRGN, FLI1, and MACROH2A2 in uEVs from longitudinal urine samples collected from the same BC patients (*n* = 5) who had experienced clinical surgeries. For SRGN, although its expression decreased after surgery in four individuals, the overall downward trend was not statistically significant (p = 0.7889, paired *t*-test) (**Figure 5G**). In contrast, the levels of FLI1 and MACROH2A2 decreased in all five individuals (p = 0.04 for FLI1 and p = 0.0435 for MACROH2A2, paired *t*-test) (**Figure 5H**,** 5I**). As radar plots illustrate, the majority of post-surgery data points were wrapped by the pre-surgery data points, which to some extent reflected the effectiveness of the clinical intervention. These results suggest that these biomarkers may be of tumor origin and are highly effective in monitoring patient prognosis.

## Discussion

BC is a common malignant tumor-bearing substantial implications for human health. Timely diagnosis of this cancer is essential to improve the survival rates of patients. In the context of diagnosis and screening, however, consideration should also be given to aspects other than sensitivity and accuracy, such as cost-effectiveness, applicability, and social acceptability among healthy cohorts. Consequently, an optimal avenue for large-scale early screening within the population still needs to be discovered. Liquid biopsy emerges as a pivotal technological stride in tumor screening and subsequent treatment. This methodology hinges upon the release of circulating tumor cells, free nucleic acids, and EVs carrying tumor-related information into the body fluids. Liquid biopsy enables the real-time and dynamic evaluation of specific molecular markers, outperforming traditional tissue biopsy that often requires invasive sample collection.

In recent years, many studies have demonstrated the burgeoning potential of EVs in cancer for clinical diagnosis, prognosis, and anticipation of therapeutic response 61,62. The quest for disease-specific EV biomarkers, particularly in cancer and other ailments, has captivated researchers, as evidenced by a surge in transcriptomic sequencing investigations of EVs. These biomarkers, which are pivotal in regulating disease progression, have emerged as promising clinical biomarkers in their capacity. RNAs in EVs not only exhibits heightened resilience to degradation but significantly offers the physiological and pathological states of the parental cells. The allure of urine-based testing lies in its potential to increase the concentration of pertinent biomarkers, concurrently minimizing interference from EVs secreted by other tumor cells due to the intimate correlation between urine and the urinary system.

In the present research, numerous transcriptomic analyses have been performed on EVs secreted by BC cell lines. These studies have undergone comprehensive and systematic analytical exploration to uncover potential diagnostic biomarkers. Notably, tumor cells represent a relatively simple environment devoid of the intricate complexities of the *in vivo* tumor microenvironment. We identified SRGN, FLI1, and MACROH2A2 in uEVs as potential biomarkers for BC detection, showing a clear distinction between healthy cohorts and early-stage BC patients. This evidence suggests their potential involvement in the early detection of BC. Moreover, our investigation reveals a significant downregulation in the expression levels of these uEV-associated biomarkers after surgery, indicating the likely secretion of SRGN, FLI1, and MACROH2A2 by tumor cellular EVs. Consequently, these findings highlight the promising prospects of these biomarkers as supplementary tools in existing clinical methodologies, potentially serving as a noninvasive assay for BC. Such an assay could offer substantial clinical value to patients by minimizing the frequency of cystoscopy, thereby enhancing their quality of life.

Multiple investigations show the oncogenic roles of SRGN, FLI1, and MACROH2A2 in various cancers. In both *in-vivo* and *in-vitro* experiments, SRGN activates the ERK pathway, stabilizes c-Myc, and up-regulates the secretion of matrix metalloproteinases to promote invasion and metastasis of esophageal squamous cell carcinoma (ESCC), and high expression of SRGN in serum of patients with ESCC is an independent prognostic biomarker of poor survival 63. The binding of SRGN to CD44 induces cytoskeletal reorganization and facilitates Src-mediated turnover of adhesion to the lesions, thereby increasing cell migration in non-small cell lung cancer 64. In Ewing sarcoma, chromosomal translocations lead to the formation of Ewing sarcoma breakpoint region 1- FLI1 (EWS-FLI1) fusion oncogene. Speckled POZ protein (SPOP) and OTU structural domain-containing protein 7A (OTUD7A) control the turnover of EWS-FLI1 protein in Ewing sarcoma. Enhancing SPOP activity and deleting OTUD7A could reduce EWS-FLI1 protein abundance and thus hinder Ewing sarcoma growth *in vitro* and *in vivo* 65. Removal of MACROH2A2 from hepatoblastoma cells could alter the transcriptional ground state of cancer cells as well as their response to inflammatory cytokines, which is related to the interaction of cancer cells with immune cells in their microenvironment 55. To our knowledge, the present study is the inaugural assessment of the diagnostic potential of uEV-derived SRGN, FLI1, and MACROH2A2 specifically in BC. This research represents an advancement in the understanding and potential clinical application of these biomarkers.

We acknowledge the limitations inherent in this study. Firstly, the relatively small sample size poses a constraint, necessitating a broader cohort study for prospective analyses to translate our findings into clinically meaningful applications. Furthermore, the procuring of pre- and post-surgery urine samples within a more extensive cohort of patients with BC presents another limitation. The roles of SRGN, FLI1, and MACROH2A2 in carcinogenesis within other cancers are established, yet their mechanisms of action in uEVs of BC remain unclear. Thus, further investigations through cellular and animal studies are imperative. Lastly, we expect the development of a more streamlined technique for isolating uEVs in urine, promising practical utility in clinical applications.

In summary, we employed transcriptomic analysis to unveil an unprecedented uEV-associated mRNA signature, offering promising prospects for BC detection, especially in the early stages. SRGN, FLI1, and MACROH2A2 have undergone successful validation within the cohort of BC patients, demonstrating exceptional potential in robustly distinguishing healthy cohorts from BC patients and presenting implications in early diagnosis and prognostication of BC. Our conjecture revolves around the prospective utilization of the three uEV-mRNA biomarkers as a potent panel for non-invasive large-scale screening for BC and envisions substantial strides in the clinical study of uEVs.

## Supplementary Material

Supplementary figures and tables.
