# Supplementary material for: Unveiling urinary extracellular vesicle mRNA signature for early diagnosis and prognosis of bladder cancer
Source: Theranostics. 2025 Jan 1;15(4):1272–84. doi: 10.7150/thno.107213 (PMC11729556; doi:10.7150/thno.107213)
Supplement: Supplementary file 1 — Supplementary figures and tables. [file thnov15p1272s1.pdf]

Supplementary Material for

Unveiling urinary extracellular vesicle mRNA signature for early diagnosis and prognosis of bladder cancer

Ning Sun,[1#] Zhaowei Zhang,[1#] Xiaoqing Yang,[2*] Jingqi Li,[1] Qiang Li,[1] Jingjing Kang,[1] Yongchun Wei,[1] Xiaoxuan Yu,[1] Rui Du,[1] Xiaoqin Hong,[1] Guangming Liu,[3] Hongmei Gao,[4*] Dingbin Liu[1*]

[1] State Key Laboratory of Medicinal Chemical Biology, Tianjin Key Laboratory of Molecular Recognition and Biosensing, Frontiers Science Center for New Organic Matter, College of Chemistry, Nankai University, Tianjin 300071, China.

[2] Tianjin Institute of Urology, the Second Hospital of Tianjin Medical University, Tianjin 300211, China.

[3] Department of Urology, Tianjin First Center Hospital, Nankai University, Tianjin 300071, China.

[4] Department of Intensive Care Unit, Key Laboratory for Critical Care Medicine of the Ministry of Health, Emergency Medicine Research Institute, Tianjin First Center Hospital, Nankai University, Tianjin 300071, China.

# These authors contributed equally to this work and should be considered co-first authors.

* Corresponding author emails:

liudb@nankai.edu.cn (Dingbin Liu);

ghm182@163.com (Hongmei Gao);

YangXiaoqing_ey@tmu.edu.cn (Xiaoqing Yang)

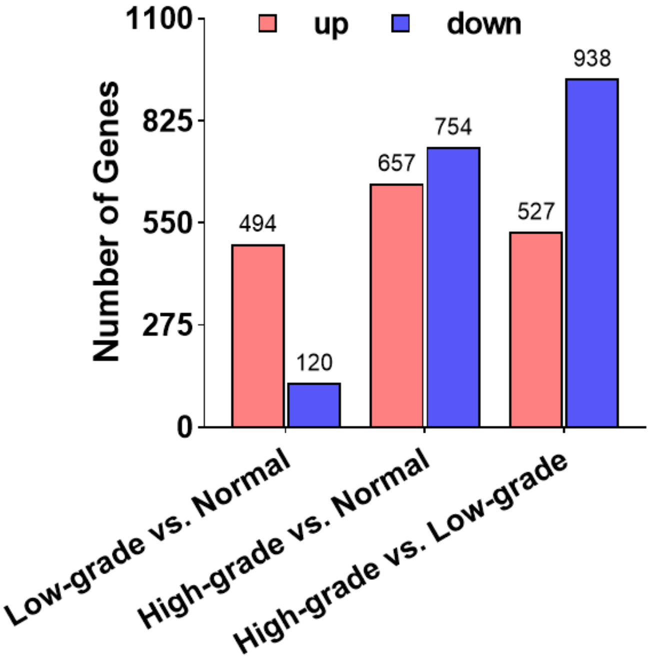

Figure S1. DEG numbers in EVs derived from the cells: low-grade BC cells relative to normal cells, high-grade BC cells relative to normal cells, and high-grade BC cells relative to low-grade cells. P-value < 0.05 and fold change (FC) > 1.5 are used to define DEGs.

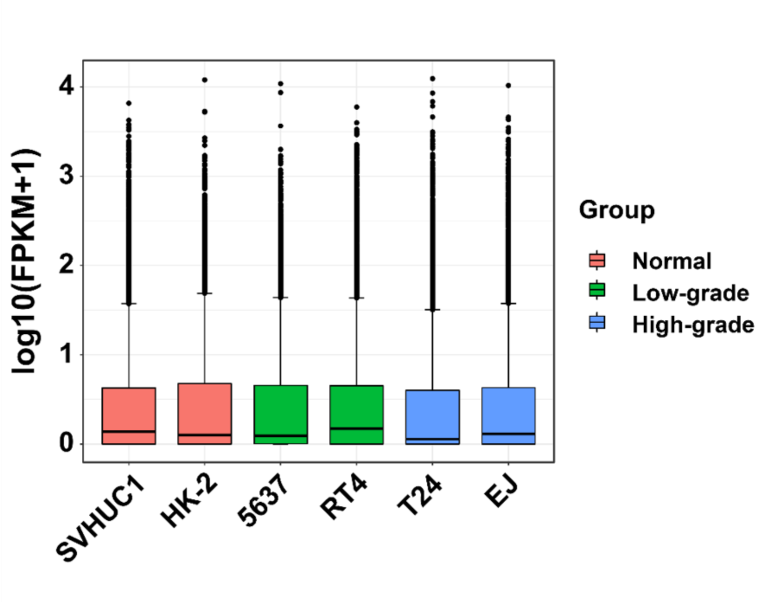

Figure S2. Analysis of gene expression levels for EVs derived from 6 cell lines.

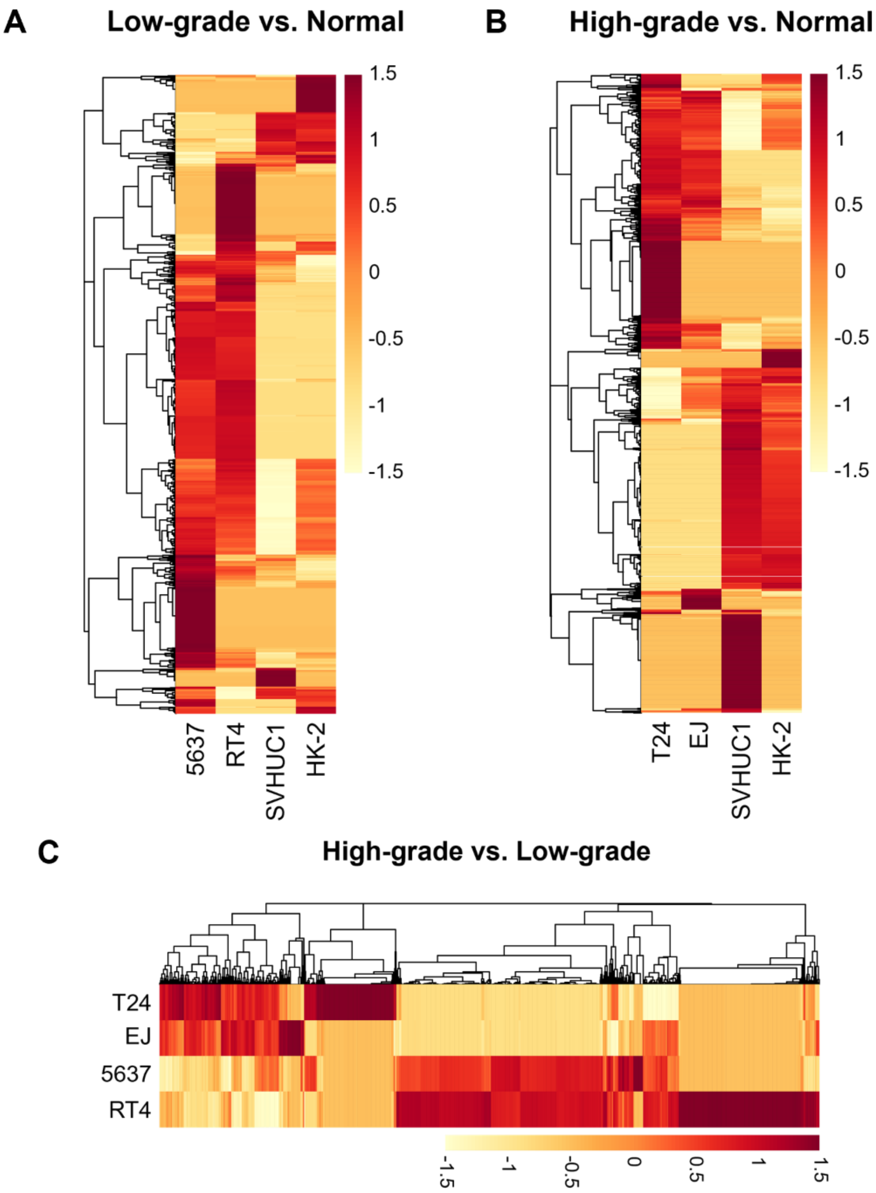

Figure S3. Hierarchical clustering analysis of proteomic data for EVs derived from the cells: (**A**) low-grade BC cells relative to normal cells, (**B**) high-grade BC cells relative to normal cells, and (**C**) high-grade BC cells relative to low-grade BC cells.

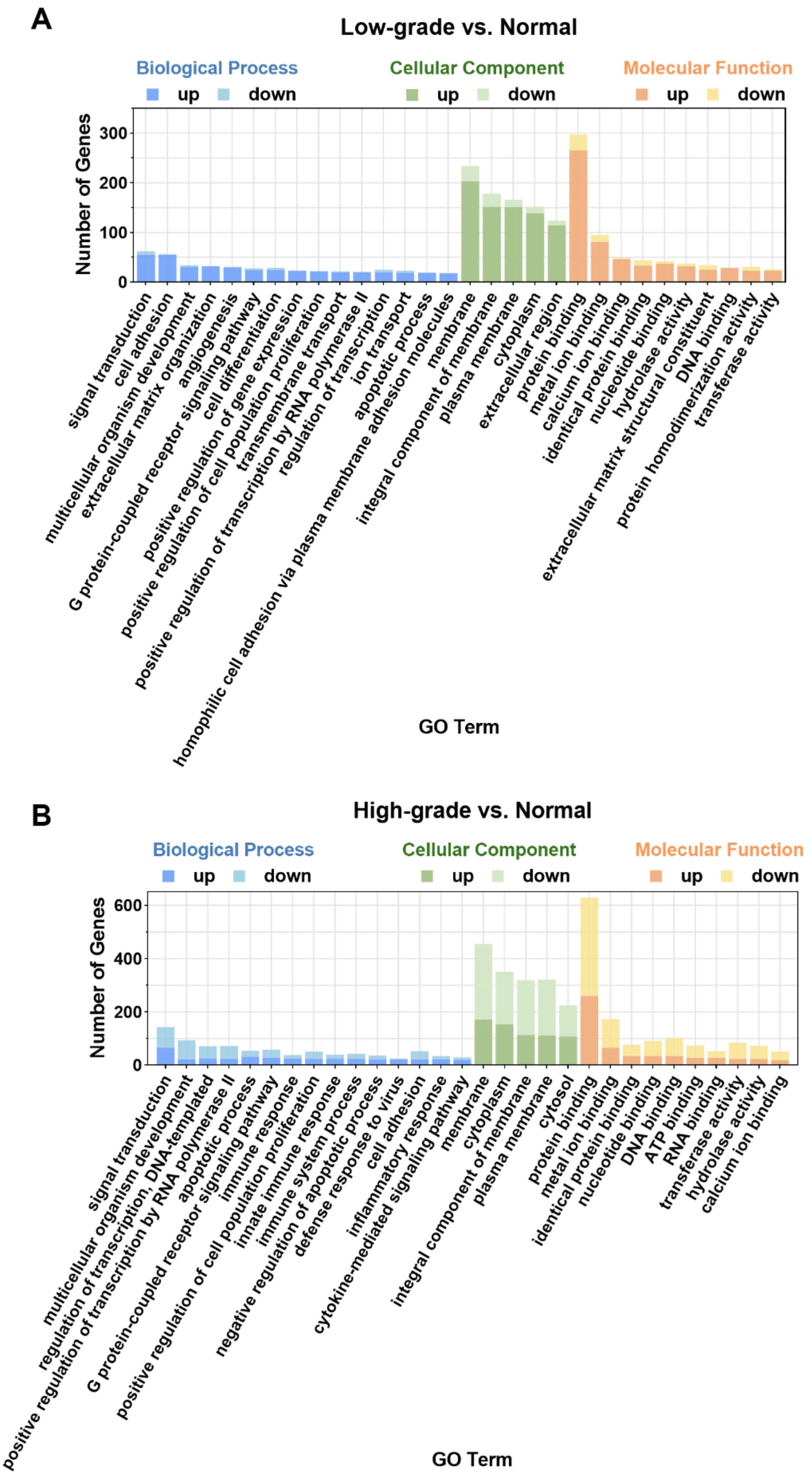

Figure S4. GO analysis based on the identified DEGs in EVs derived from the cells: (**A**) low-grade BC cells relative to normal cells and (**B**) high-grade BC cells relative to normal cells.

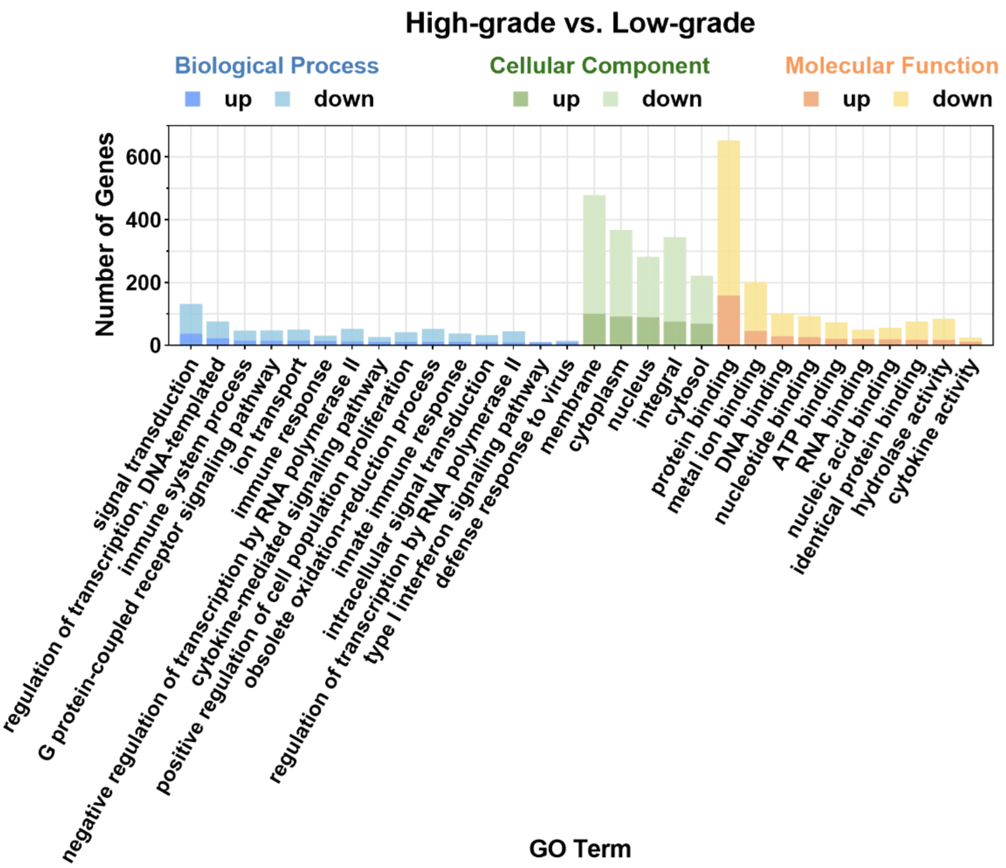

Figure S5. GO analysis based on the identified DEGs in EVs derived from the cells (high-grade BC cells relative to low-grade BC cells).

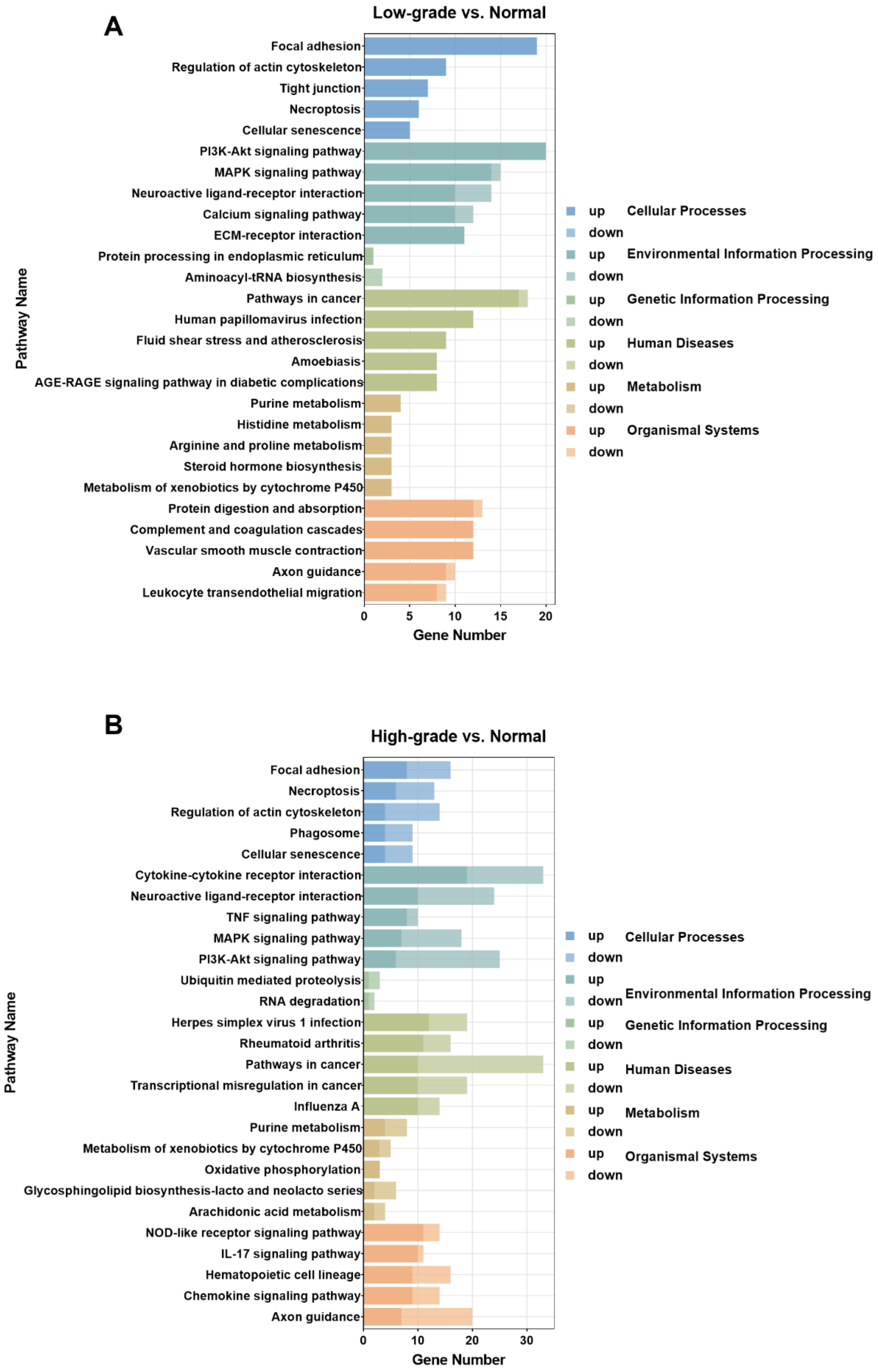

Figure S6. KEGG analysis based on the identified DEGs in EVs derived from the cells: (**A**) low-grade BC cells relative to normal cells and (**B**) high-grade BC cells relative to normal cells.

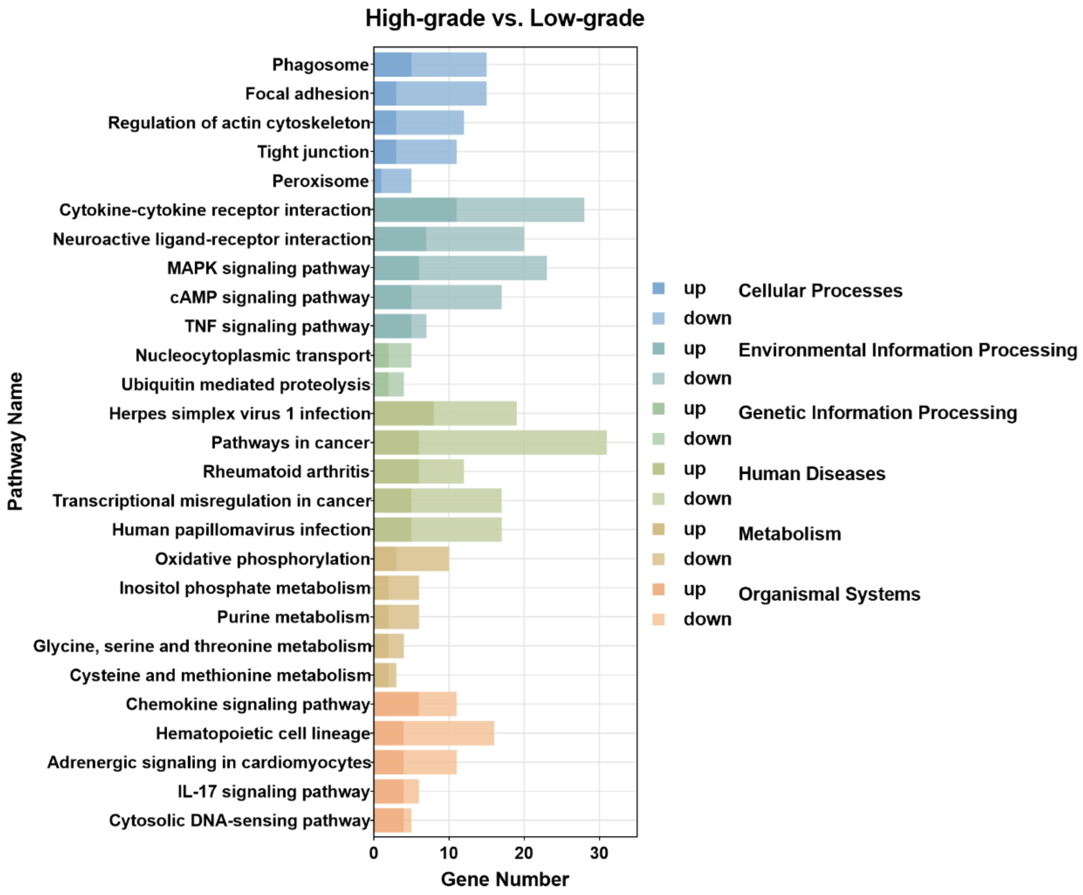

Figure S7. KEGG analysis based on the identified DEGs in EVs derived from the cells (high-grade BC cells relative to low-grade BC cells).

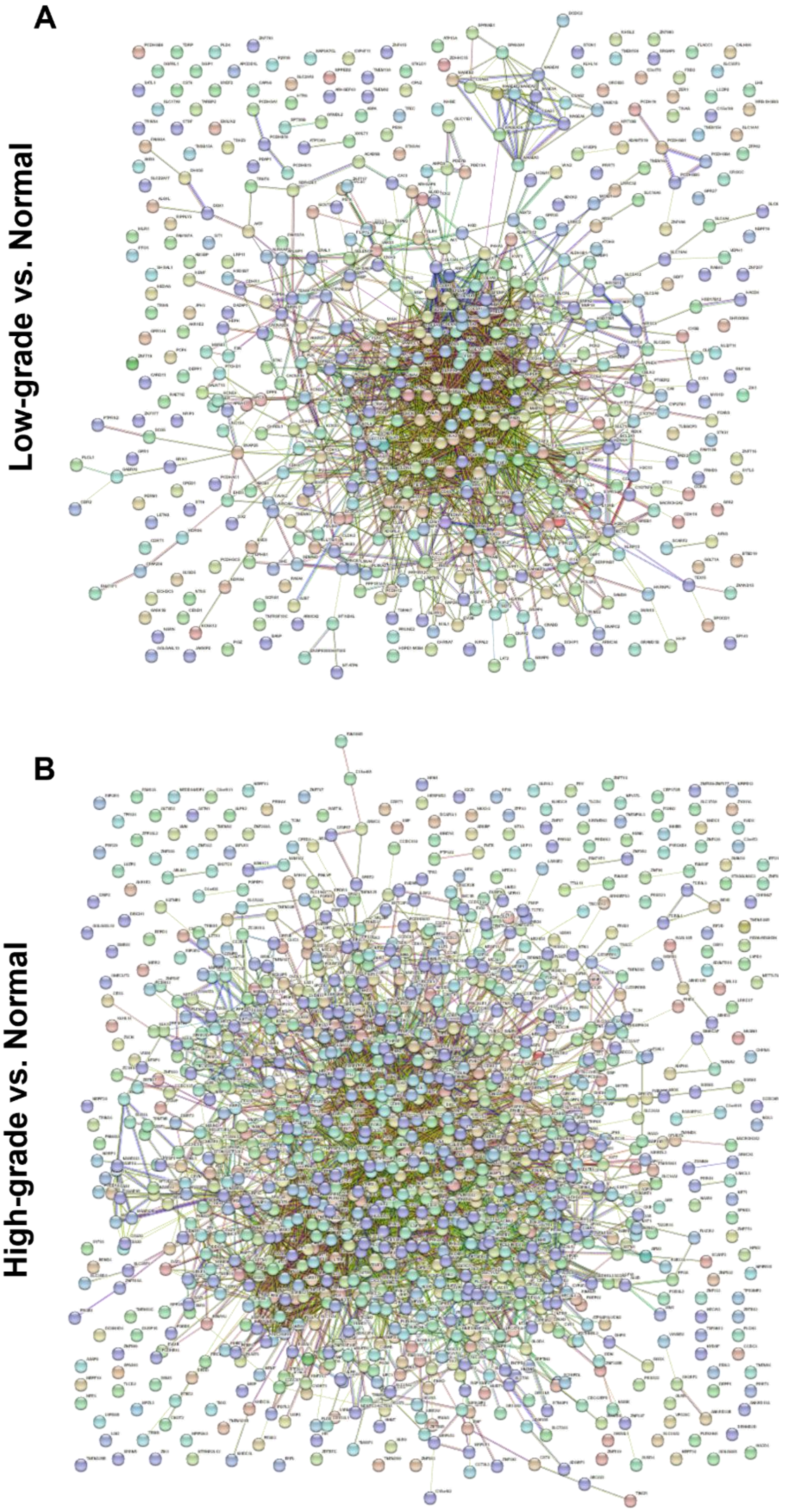

Figure S8. PPI network diagrams of the identified DEGs in EVs derived from the cells: (**A**) low-grade BC cells relative to normal cells and (**B**) high-grade BC cells relative to normal cells.

High-grade vs. Low-grade

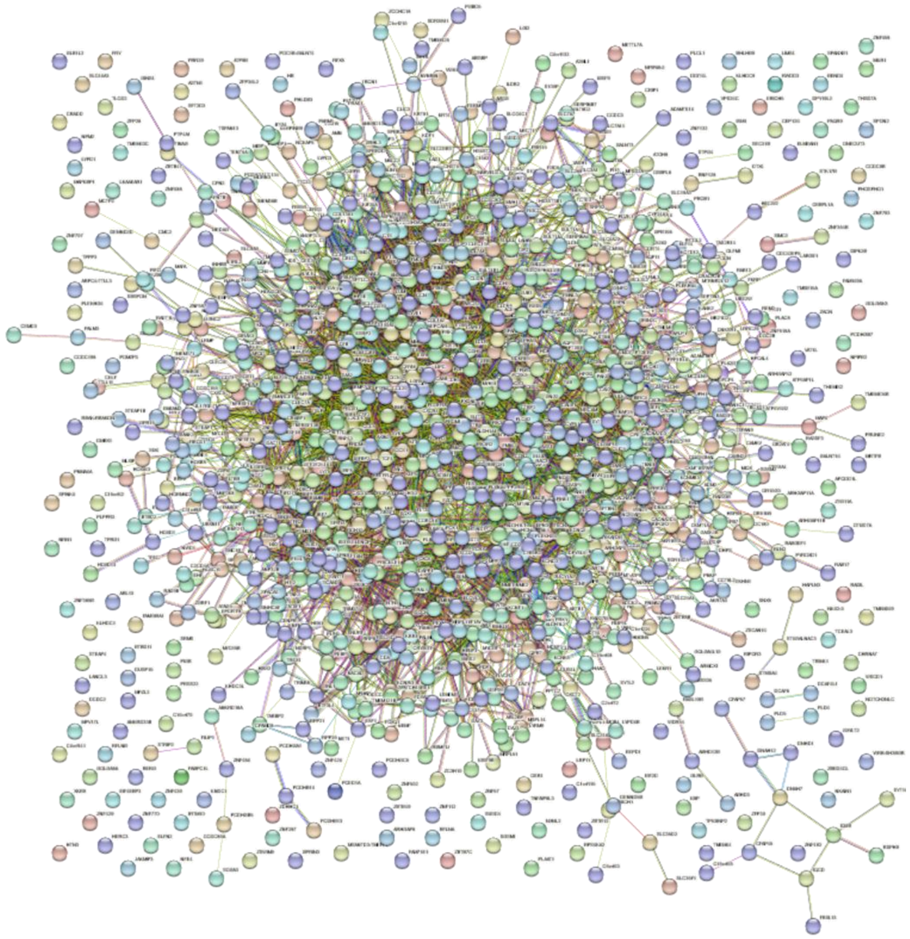

Figure S9. PPI network diagrams of the identified DEGs in EVs derived from the cells (high-grade BC cells relative to low-grade BC cells).

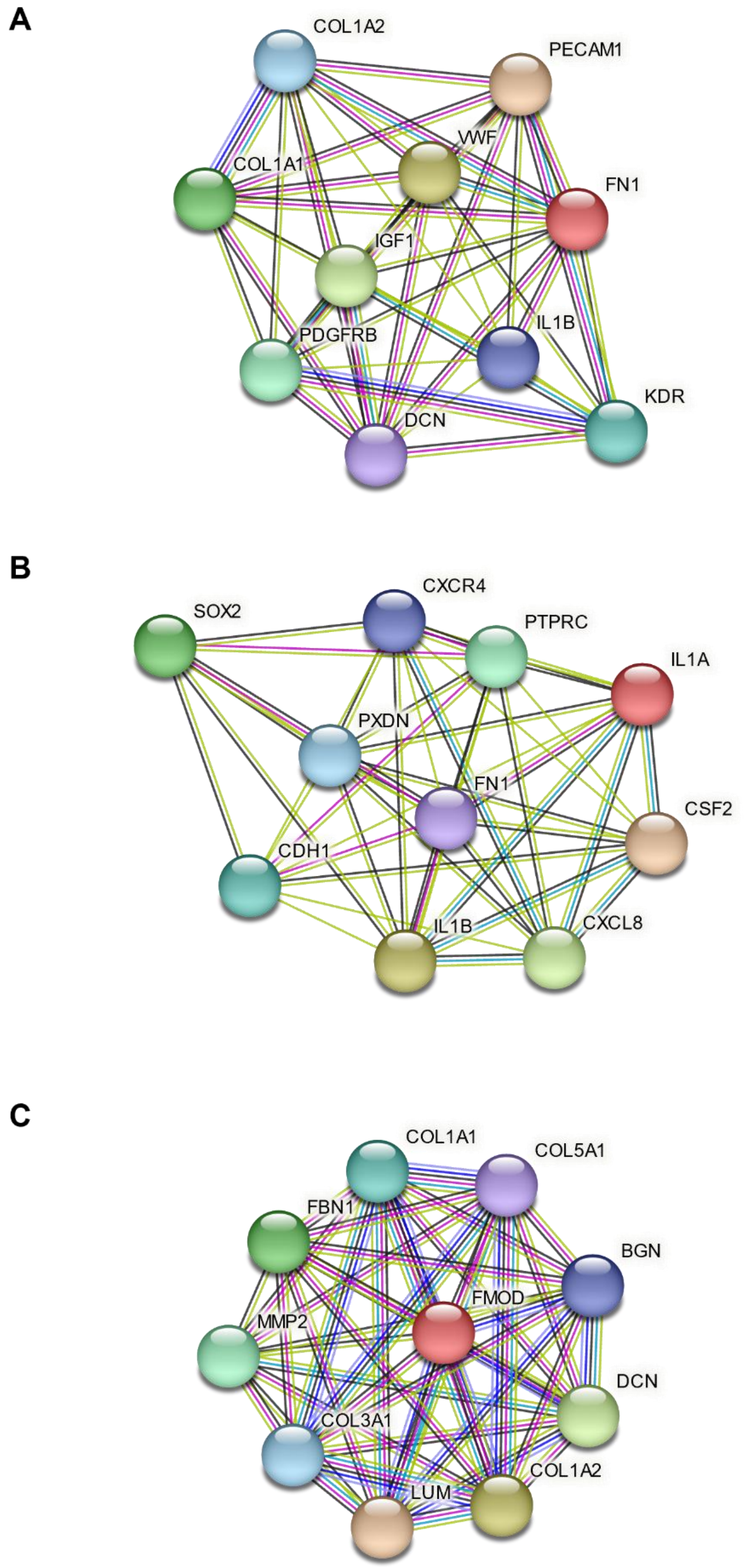

Figure S10. Hub genes generated from PPI network in EVs derived from the cells: (**A**) low-grade BC cells relative to normal cells, (**B**) high-grade BC cells relative to normal cells, and (**C**) high-grade BC cells relative to low-grade cells.

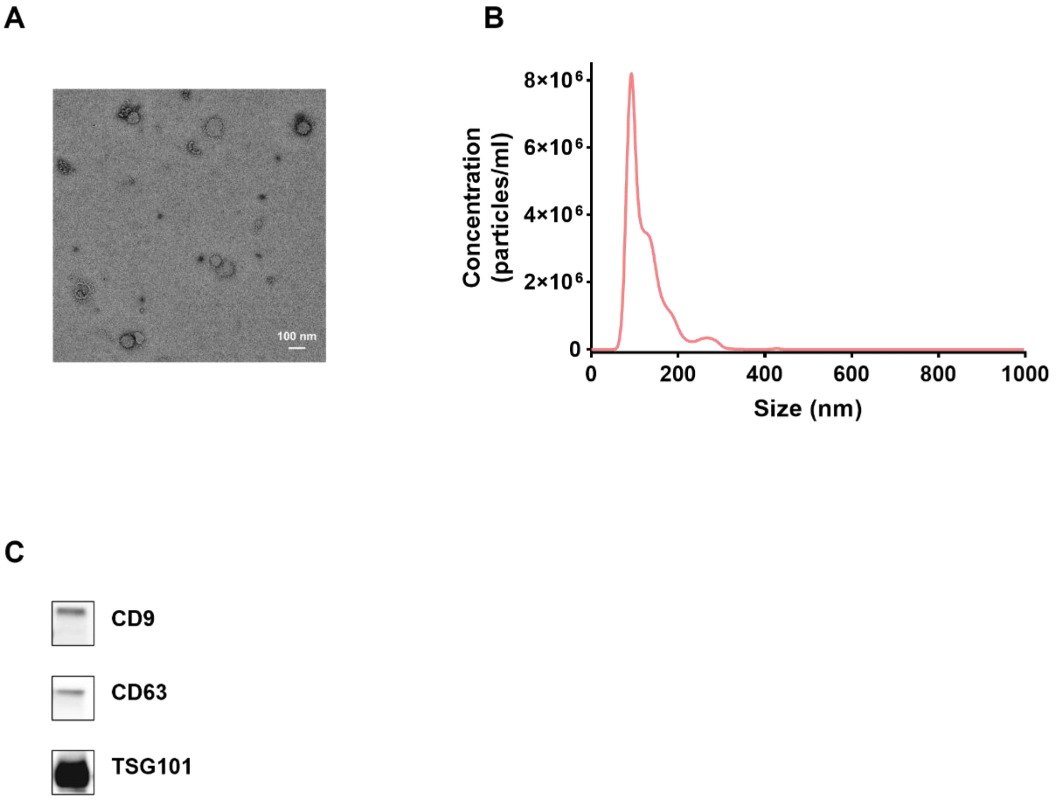

Figure S11. Characterization of uEVs derived from clinical urine sample by (**A**) cryo-TEM, (**B**) NTA, and (**C**) WB.

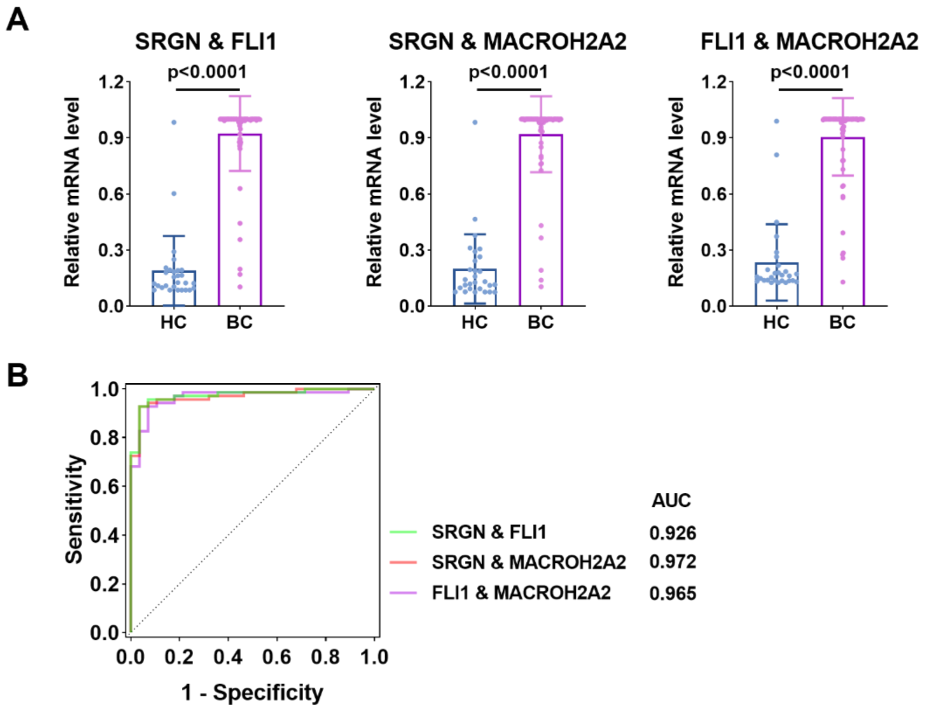

Figure S12. Combining two biomarkers to distinguish HC and BC. (**A**) Scattering plots of the mRNA expression levels of the weighted sum of the biomarkers two by two, SRGN and FLI1, SRGN and MACROH2A2, and FLI1 and MACROH2A2, respectively, in uEVs collected from HC and BC patients. (**B**) ROC curves of the weighted sum of the biomarkers two by two for BC diagnosis. The weighted sum of the three markers was calculated by logistic regression algorithm.

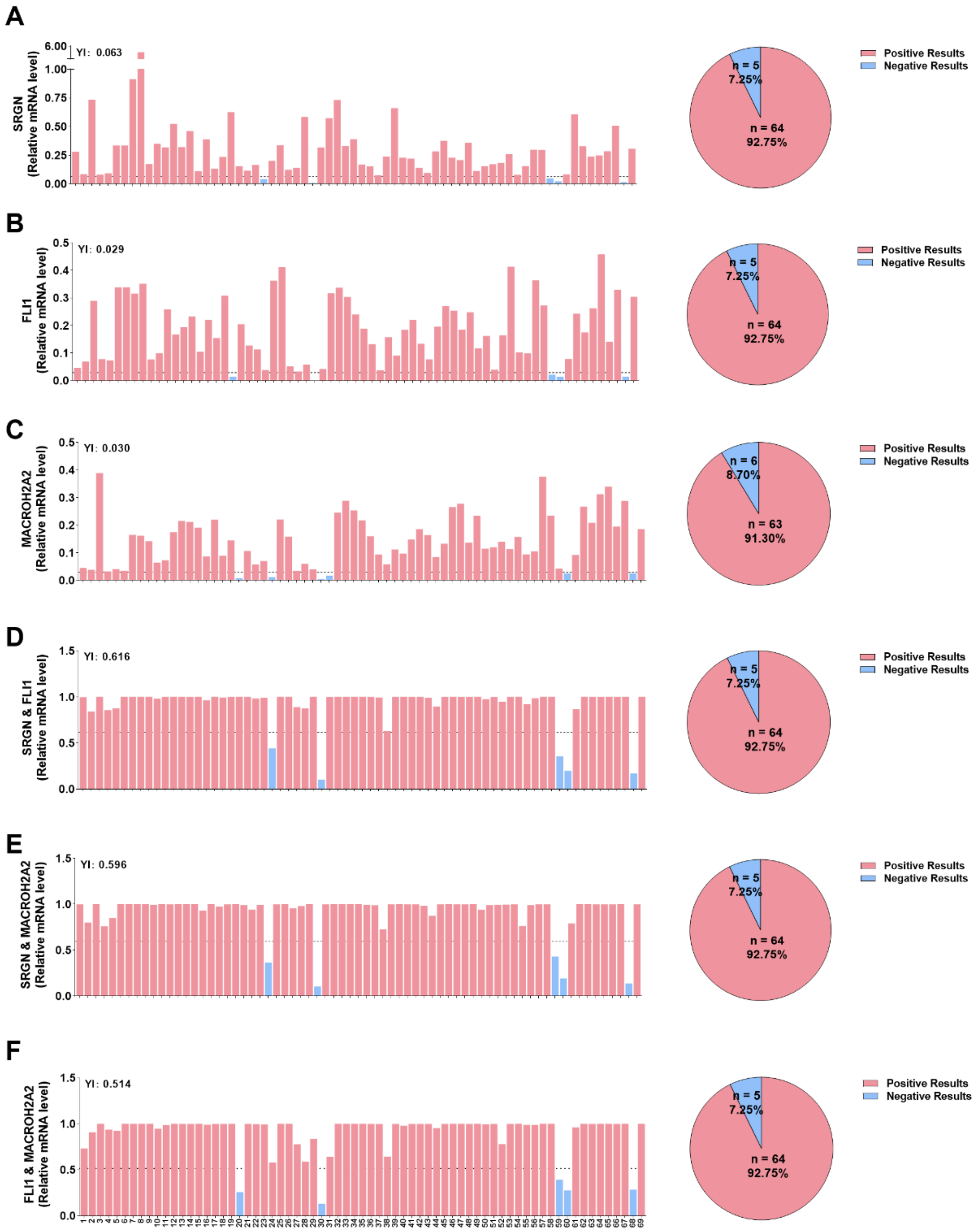

Figure S13. BC subjects were divided into two subgroups of high and low mRNA expression level by the weighted sum of biomarkers. (**A**) SRGN, (**B**) FLI1, (**C**) MACROH2A2, (**D**) SRGN & FLI1, (**E**) SRGN & MACROH2A2, and (**F**) FLI1 & MACROH2A2. Cut-off values were determined using YI.

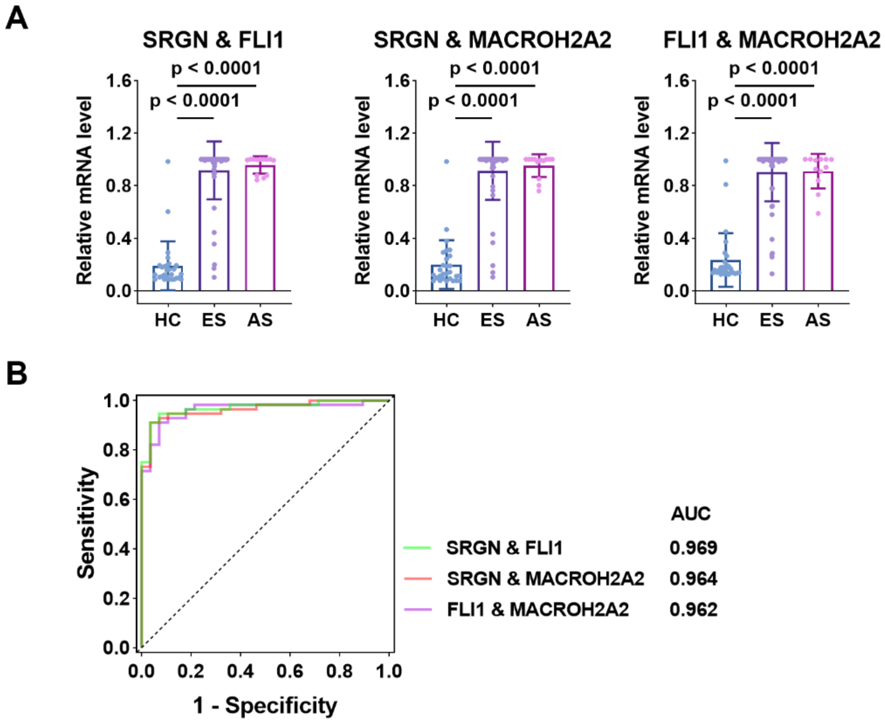

Figure S14. Combining two biomarkers to distinguish HC and early-stage BC. (**A**) Scattering plots of the mRNA expression levels of the weighted sum of the biomarkers two by two, SRGN and FLI1, SRGN and MACROH2A2, and FLI1 and MACROH2A2, respectively, in uEVs collected from HC, early-stage BC patients, and advanced-stage BC patients. (**B**) ROC curves of the weighted sum of the biomarkers two by two for early-stage BC diagnosis. The weighted sum of the three markers was calculated by logistic regression algorithm.

Table S1. The primer sequences used for RT-qPCR.

Genes	Forward Primer (5'-3')	Reverse Primer (5'-3')
**FAM83A**	GGCCCTAAGGGACTGGACT	CACAGTGGCGCTGGATTTTT
**SRGN**	GATCTGGGAGTGGCTTCCTAACG	CTGAGGGCAGATTCCTGTCAAGAG
**FLI1**	ATGTGAGGCAATGGCTGGAGTG	CTTCCGTGTTGTAGAGGGTGGTG
**THBD**	AGTGGGTTACGGGAGACAACAAC	CTCGCAGAGGAAGCCATCGG
**PTPRN2**	GAAGAGTGTGCTTGTCATCTGC	AATGCCAAACGTGCCTTGTG
**ZNF883**	TCAGCCGCAGCACACACC	TGGGTTCTCTGATGTCGGATTAGTG
**MACROH2A2**	GGGAGGCTGATGCGTTATCTGAAG	CCGCTGCCAGGTACTCAATGAC
**IL1B**	GGACAGGATATGGAGCAACAAGTGG	TCATCTTTCAACACGCAGGACAGG
**GAPDH**	AAGGAGTAAGACCCCTGGACC	ACTGTGAGGAGGGGAGATTCA

Table S2. Summary of the clinical cohorts.

ID	Sex	Age	Tumor stage	ID	Sex	Age	Tumor stage
**1**	male	88	T2	**36**	male	68	T1
**2**	male	84	T3	**37**	male	68	T1
**3**	female	83	T3	**38**	male	68	T1
**4**	male	82	T1	**39**	male	68	T1
**5**	female	82	T1	**40**	male	68	T1
**6**	female	79	T1	**41**	male	67	T3
**7**	male	78	Ta	**42**	male	67	Ta
**8**	male	78	T1	**43**	male	67	Ta
**9**	male	76	T3	**44**	male	67	Ta
**10**	female	76	Ta	**45**	male	66	Ta
**11**	female	75	T2	**46**	male	66	Ta
**12**	male	75	T1	**47**	male	66	Ta
**13**	male	74	T1	**48**	male	65	T3
**14**	male	74	T1	**49**	male	65	T3
**15**	male	74	Ta	**50**	male	65	T2
**16**	female	73	Ta	**51**	male	65	T2
**17**	male	73	T1	**52**	male	64	Ta
**18**	male	73	T1	**53**	male	64	T1
**19**	female	73	Ta	**54**	male	64	Ta
**20**	male	72	T1	**55**	male	63	T1
**21**	male	72	T2	**56**	male	63	T1

**22**	male	72	T1	**57**	male	62	T1
**23**	male	72	Ta	**58**	female	60	Ta
**24**	male	72	T1	**59**	male	59	Ta
**25**	male	71	T1	**60**	male	59	T1
**26**	male	71	Ta	**61**	female	58	T1
**27**	female	70	T1	**62**	female	58	T1
**28**	female	70	Ta	**63**	male	55	T1
**29**	male	70	T1	**64**	male	52	T1
**30**	male	70	Ta	**65**	female	50	T1
**31**	male	70	T1	**66**	male	50	Ta
**32**	male	70	T1	**67**	male	43	Ta
**33**	female	69	T3	**68**	male	38	Ta
**34**	female	69	T3	**69**	female	29	Ta
**35**	male	69	Ta				
**1**	male	72	n/a	**15**	female	48	n/a
**2**	female	71	n/a	**16**	male	43	n/a
**3**	male	69	n/a	**17**	female	41	n/a
**4**	male	69	n/a	**18**	male	35	n/a
**5**	male	67	n/a	**19**	male	35	n/a
**6**	female	66	n/a	**20**	male	32	n/a
**7**	male	65	n/a	**21**	male	30	n/a
**8**	male	64	n/a	**22**	female	29	n/a
**9**	female	60	n/a	**23**	female	27	n/a
**10**	male	58	n/a	**24**	female	26	n/a
**11**	male	56	n/a	**25**	female	25	n/a
**12**	female	54	n/a	**26**	male	25	n/a
**13**	male	52	n/a	**27**	female	24	n/a
**14**	male	50	n/a	**28**	female	24	n/a

Table S3. ROC curve analysis results of mRNA combinations for BC diagnosis.

	AUC	95%CI	Sensibility	Specificity
**SRGN**	0.9695	0.9380-1.000	92.8	96.4
**FLI1**	0.9658	0.9308-1.000	92.8	92.9
**MACROH2A2**	0.9260	0.8736-0.9784	91.3	82.1
**SRGN & FLI1**	0.9731	0.9436-1.000	92.8	96.4
**SRGN & MACROH2A2**	0.9689	0.9374-1.000	92.8	96.4
**FLI1 & MACROH2A2**	0.9648	0.9287-1.000	92.8	92.9
**SRGN & FLI1 & MACROH2A2**	0.9731	0.9436-1.000	92.8	96.4

Table S4. ROC curve analysis results of mRNA combinations for early-stage BC diagnosis.

	AUC	95%CI	Sensibility	Specificity
**SRGN**	0.9649	0.9289-1.000	94.6	92.9
**FLI1**	0.9630	0.9248-1.000	91.1	92.9
**MACROH2A2**	0.9260	0.8702-0.9818	83.9	92.9
**SRGN & FLI1**	0.9694	0.9358-1.000	94.6	92.9
**SRGN & MACROH2A2**	0.9643	0.9281-1.000	91.1	96.4
**FLI1 & MACROH2A2**	0.9617	0.9219-1.000	91.1	92.9
**SRGN & FLI1 & MACROH2A2**	0.9694	0.9358-1.000	94.6	92.9

Table S5. PCR reaction system.

Reagent	50 mL Reaction system	Final concentration
**2 × SGExcel FastSYBR Mixture**	25 μL	1 ×
**Forward Primer, 10μM**	1 μL	0.2 μM
**Reverse Primer, 10μM**	1 μL	0.2 μM
**cDNA**	2 μL	-
**RNase-Free ddH2O**	21 μL	-

Table S6. PCR response procedures.

Procedure	Temperature / ºC	Time / s	Cycle number
**Predenaturation**	95	180	-
**Denaturation**	95	5	40
**Annealing / Extension**	60	20	
	95	10	
**Melting analysis**	65	60	-
	97	1	
